# Intraretinal Electrophysiology and Resistivity Profiles of WT and RCS Rat Retina

**DOI:** 10.3390/s25123765

**Published:** 2025-06-16

**Authors:** Marie Jung, Antje Willuweit, Viviana Rincón Montes

**Affiliations:** 1 Institute of Biological Information Processing (IBI-3), Bioelectronics, Forschungszentrum Jülich, 52428 Jülich, Germany; 2 Department of Physics, RWTH Aachen University, 52056 Aachen, Germany; 3 Institute of Neuroscience and Medicine (INM-4), Medical Imaging Physics, Forschungszentrum Jülich, Leo-Brandt-Str., 52428 Jülich, Germany

**Keywords:** wildtype rat, Royal College of Surgeons rat, retinal degeneration, retinal impedance, electrophysiology

## Abstract

Retinal prostheses have been utilized in the treatment of blindness resulting from retinal degeneration. However, they have not met patient expectations, leading to market withdrawals. As a result, research continues to focus on improving visual perception, such as by modeling retinal neural activation. The retina’s electrical resistivity profile is key, as it influences the current spread during electrical stimulation. To advance efficient stimulation parameters, more data on the electrical properties of the retina in both its healthy and diseased state is needed. While this question has been addressed in mouse models, few data are available from rat models, whose bigger size is advantageous for many applications. To address this knowledge gap, we used flexible penetrating microelectrode arrays to measure intraretinal impedance and electrophysiological activity in retinas from both healthy (WT) and diseased RCS rats, an established model of retinal degeneration. Consequently, we calculated resistivity profiles, consistent with previous mouse retina findings, and correlated them with spontaneous spiking activity. Hence, both impedance and electrophysiological measurements across retinal depths are demonstrated as valuable tools to identify the optimal stimulation depth and simulate the electric field spread during electrical stimulation, which is particularly useful for the development of retinal prostheses. These findings demonstrate that resistivity changes in the degenerated retina significantly impact stimulation protocols and electric field propagation.

## 1. Introduction

Retinal prostheses have made the transition from the laboratory to the clinic and have restored partial vision to blind people affected by retinal degeneration. Despite this progress, implants have had difficulty meeting patient expectations and CE-marked devices have been withdrawn from the market [1]. Ongoing research is therefore aimed at improving the quality of visual perception. For efficient electrical stimulation of the degenerated retina, a key aspect is modeling and simulating retinal neural activation through electrical stimulation in order to understand spatial patterns [2,3,4,5]. The retina is a neural layer located at the back of the eye that pre-processes and transduces visual information into electrical signals. These signals are transmitted through the optic nerve to the brain, where they undergo further processing to form visual perception. The layered architecture and anisotropic anatomy of the retina (e.g., neurons more densely packed in the inner nuclear layer—INL) gives rise to a spatially varying electrical resistivity, forming an intrinsic resistivity profile, a depth-dependent distribution of resistivity across retinal layers [6,7,8]. This resistivity profile plays a critical role in the context of retinal prostheses, as it influences the intraretinal current flow and the resulting potential distribution, therefore influencing the selection of electrical stimulation parameters during stimulation of the retina.

Rats and mice are often the models of choice for medical device validation because they offer several advantages over other species. They reproduce rapidly, and their size and adaptability make them easy to care for and to work with. Both species have been used in research for the last century, and several strains are now well characterized genetically and physiologically. Some strains develop diseases spontaneously, such as the *rd10* mouse [9] or the Royal College of Surgeon (RCS) rat [10] strains, models for the degenerative retinal disease of retinitis pigmentosa. However, there are some cellular differences that result from species-specific adaptations to the environment and behavioral factors. For example, rodents are nocturnal and therefore their retina is rod-dominated [11]. Due to the superior size of the rat eye compared to that of a mouse, rats are an animal model of interest for retinal applications, such as for retinal prosthetic devices [12]. Previous measurements of retinal resistivity were mostly limited to healthy retina, including studies of the retinal resistivity profile of birds [8], frogs [13], chickens [7], rats [7], and mice [6]. While there are publications on the resistivity profile of diseased retina of mice [6], to the best of our knowledge there is currently no study on the resistivity profile of a diseased rat retina. Mapping resistivity profiles across species provides critical insights into the design and optimization of retinal prostheses. Detailed resistivity profiles enable more accurate modeling and simulation of electric field distributions within the retina, which is essential for assessing optimal stimulation position, predicting neural activation patterns, and improving the spatial precision and efficacy of prosthetic stimulation.

Resistivity measurements in neural tissue are typically obtained by applying mathematical models to electrochemical impedance spectra recorded using invasive microelectrodes, such as microwires, glass micropipettes, or penetrating microelectrode arrays (MEAs). Common analytical approaches include the least squares method and the peak resistance frequency (PRF) method. The PRF method, originally described by Mercanzini et al. [14] and further analyzed by Weiland et al. [15], is widely used due to its rapid computation and adequate precision [6,7,16]. While less commonly employed, other techniques, such as electrical impedance tomography, offer non-invasive alternatives. However, this requires a more complex experimental setup, typically involving planar MEAs in combination with optical coherence tomography to estimate retinal depth [17].

To further characterize the electrical properties of the diseased rat retina, we utilized flexible penetrating multisite microelectrode arrays to generate a resistivity profile of the retinal layers and correlate this with electrophysiological activity in both healthy (WT) and diseased explanted RCS rat retinas. To achieve this, we utilized flexible penetrating probes to measure impedance at different retinal depths and subsequently extracted the resistivity profiles of the retinal layers. Simultaneously, electrophysiological recordings were conducted at each intraretinal depth to correlate spontaneous spiking activity (action potentials) and noise levels with resistivity values. These findings provide insights that enable the fine-tuning of electrode positioning within the retina to optimize electrophysiological recordings and electrical stimulation of the retinal network. Furthermore, the spread of the electric field was simulated using COMSOL Multiphysics version 5.6 to examine how the resistivity profiles of healthy and diseased retinas influence electrical stimulation.

## 2. Material and Methods

### 2.1. Design and Fabrication of Intraretinal Probe

**Figure 1:**
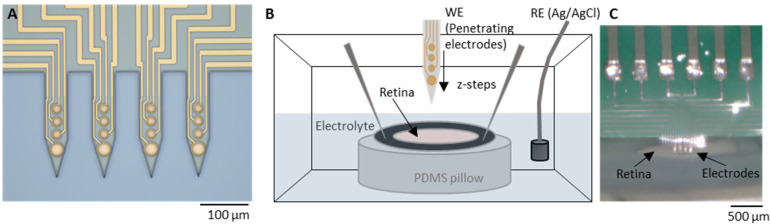
Experimental setup. (**A**) Light microscopy pictures of a penetrating flexible intraretinal probe. The probe consists of four shanks, each 225 µm long and 50 µm wide. Each shank contains four electrodes: three with a diameter of 15 µm and one bottom electrode with a diameter of 25 µm (dimensions in µm as marked in the image). (**B**) The perfusion chamber with the explanted retina, with retinal ganglion cells (RGCs) facing upwards, was immersed in a fresh oxygenated medium. The retina explant was attached to a hollow filter paper and fixed on a polydimethylsiloxane (PDMS) substrate with insect pins. (**C**) Light microscopy picture of an intraretinal probe at the surface of the retina from the epiretinal side.

We employed flexible, penetrating, multi-shank, multisite intraretinal probes with a comb-like design, as previously introduced for intraretinal applications. Known as flexible bidirectional microelectrode arrays (flexible BiMEAs), to enable the simultaneous electrical recording and stimulation across retinal depths [18,19], each probe comprises four shanks, each measuring 225 µm in length. Each shank has a total thickness of 10 µm, provides a sensing depth of 106 µm, and integrates four electrodes: three recording electrodes with a diameter of 15 µm and one stimulating electrode with a diameter of 25 µm (Figure 1A). Based on parylene-C (PaC) as the main structural material, the design of the intraretinal probes provides a cross-sectional geometry and length that ensures mechanical stability for insertion in the retina without the need of additional insertion aids, as previously reported [18,19]. In addition, these probes provide close coupling to target cells, lowering stimulation thresholds [20], while their flexible design minimizes tissue damage upon insertion compared to rigid probes [18].

The fabrication of flexible BiMEAs, from here onwards referred to as intraretinal probes, involved the deposition of two flexible thin film layers and one metal layer. Microfabrication of the probes was carried out at the Helmholtz Nano Facility (HNF) at the *Forschungszentrum* Jülich [21] following previously reported microfabrication methods [18,22]. For the fabrication of intraretinal probes, 5 µm of PaC was first deposited on a host silicon (Si) wafer via chemical vapor deposition (CVD) using a PDS 2010 Labcoater 2 (Specialty Coating Systems Inc., Indianapolis, IN, USA), corresponding to 10 g of PaC dimer and a process vacuum pressure of 25 mTorr. Subsequently, the metal contact pads, feedlines, and electrodes were patterned. This was accomplished by spin-coating LOR3B (MicroChemicals GmbH, Ulm, Germany) at 3000 rpm for 30 s with a ramp of 500 rpm/s and a subsequent soft bake for 5 min at 150 °C. Then, AZ nLof 2020 (MicroChemicals GmbH, Germany) was spin-coated at 3000 rpm for 30 s with a ramp of 500 rpm/s followed by a soft-bake for 1 min at 110 °C. The photoresist was then exposed at 40 mJ/cm^2^ with a mask aligner (MA8/BA8, Süss, Garching, Germany). Subsequently, a post-exposure bake step at 110 °C for 2 min on a direct contact hot plate was conducted, followed by a developing step in AZ 326 MIF (MicroChemicals GmbH, Ulm, Germany) for 30–35 s and a cleaning step in deionized water. Subsequently, the wafer was evaporated with a metal stack comprising 20/100/10 nm of Ti/Au/Ti, utilizing an electron-beam assisted evaporation machine (Balzer PLS 570, Pfeiffer Vacuum GmbH, Aßler, Germany). Subsequently, a metal lift-off process was conducted in an acetone bath for a period of 2.5–3 h, followed by a bath of AZ 326 MIF for 5 min and rinsing in water.

Then, a second flexible PaC layer with a thickness of 5 μm was deposited, as described previously. This layer served as the passivation of the feedlines. To remove PaC at the electrodes and contact pad openings and outline the shape of the probes, an etch mask was patterned on the final PaC layer by spin-coating two layers of the photoresist AZ 10XT (MicroChemicals GmbH, Ulm, Germany). First, the photoresist was spun at 2400 rpm for 60 s with a ramp of 500 rpm/s, followed by a soft bake of 80 s at 110 °C. During the second spin-coating step, a speed of 2100 rpm for 60 s with a ramp of 500 rpm/s was used, followed by a soft bake of 160 s at 110 °C. Then the wafer was exposed with a dose of 1900 mJ/cm^2^ using the mask aligner. This was followed by a developing step of 6–8 min using AZ400K in a dilution of 1:4 with deionized water. Following the patterning of the etch mask, a reactive ion etching (RIE) step was conducted using an oxygen/carbon tetrafluoride (O_2_/CF_4_) gas mixture of 36/4 sccm, respectively, with radio frequency (RF) and inductively coupled plasma (ICP) powers of 50/500 W, with the objective of etching PaC. A second RIE step was performed to etch the top 10 nm Ti layer using an O_2_/Ar gas mixture of 20/20 sccm with an RF power of 150 W for 75 s. Following RIE, the etch mask was stripped using AZ 100 remover (MicroChemicals GmbH, Ulm, Germany) and rinsed with isopropanol.

Next, the intraretinal probes were detached from the host silicon wafer using droplets of water. Subsequently, the probes were bonded to a customized 16-channel printed circuit board (PCB) via flip-chip bonding. To improve the packaging stability, the probe-PCB interface was coated with 1:10 PDMS and subsequently cured at 120 °C for 30 min. To reduce the impedance of the Au electrodes and render them suitable for neuronal recordings [23], PEDOT/PSS (poly (3,4-ethylenedioxythiophene: poly(4-styrenesulfonate)) was electrodeposited. Here, an EDOT/PSS solution was prepared with 3,4-Ethylenedioxythiophene (EDOT) and poly (sodium 4-styrenesulfonate) (PSS) with a 0.1% (*w*/*v*) and 0.7% (*w*/*v*) concentrations in deionized water. Before electrodeposition, the probes were first subjected to electrochemical cleaning in 1× PBS (phosphate buffered solution) at room temperature by applying 10 cyclic voltammetry cycles to all electrodes using a sweep rate of 100 mV/s and potential limits between −0.6 and 0.9 V versus an Ag/AgCl reference electrode. Then, the surface of the electrodes was activated with O_2_ plasma using a pressure of 0.8 mbar and a power of 80 W for 3 min. The electrochemical polymerization of the EDOT/PSS on the Au-based electrodes was then performed via chronoamperometry using a constant potential of 1 V for 20 s.

### 2.2. Electrode Characterization

**Figure 2:**
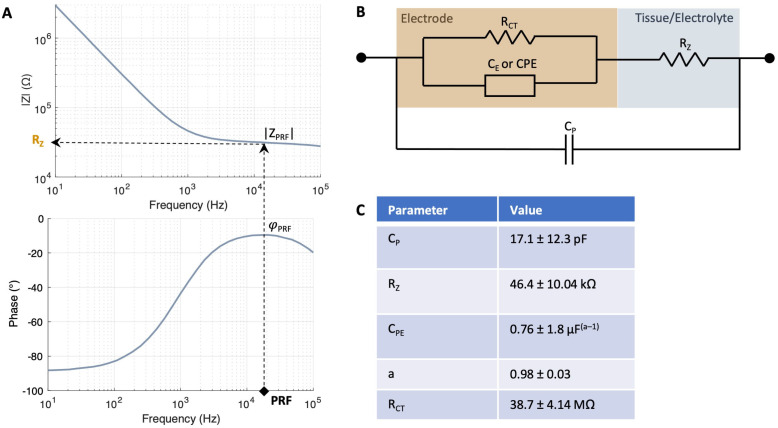
Impedance model of electrode–tissue interface. (**A**) PRF method to identify the frequency at which the impedance of the interface is most resistive, i.e., where the phase *φ_PRF_* is closest to zero degrees. Subsequently, the magnitude of the impedance at this phase |*Z_PRF_*| is registered. (**B**) The electrode–tissue interface model employed for characterizing the electrode and measuring tissue resistance is a modified Randles model. The model comprises a double layer capacitance *C_E_* represented by a constant phase element (*C_PE_*), a charge transfer resistance *R_CT_* and a tissue/electrolyte resistance *R_Z_*, which changes at each *z*-location within retina. (**C**) Values of the impedance fit measured with *N* = 7 electrodes with a diameter of 25 µm in Ames’ medium. The goodness of fit (${Χ}^{2}/|Z|$) was 7.62 ± 5.0.

Electrochemical impedance spectroscopy (EIS) measurements (Figure 2A) were carried out to characterize the electrodes using a three-electrode setup with an Ag/AgCl reference and a Pt counter electrode immersed in a 0.1 M phosphate buffered saline solution (1xPBS). The measurements were performed using a VSP-300 potentiostat (BioLogic Science Instruments, Seyssinet-Pariset, France) with a 10-mV excitation signal within a frequency range of 1 Hz–1 MHz. The impedance fit (Figure 2B,C) was performed with the impedance data using the EC-lab (BioLogic Science Instruments, Seyssinet-Pariset, France) software version v11.30. The thermal noise was calculated as follows:

(1) $$vt={\left(4k_{b}T\int_{f_{1}}^{f_{2}}Re\left(Z\right)\;df\right)}^{\frac{1}{2}}$$

In Equation (1), the Boltzmann-constant is *k_b_*, the absolute temperature *T* = 300 K, and *Re*(*Z*) is the real part of the impedance. The thermal noise was calculated within the frequency band of 300 Hz to 3000 kHz.

### 2.3. Retina Preparation and Insertion Protocol

Retinas were explanted from healthy wildtype (WT) Wistar (file number 81-02.04.2018.A190) and diseased Royal College of Surgeons (RCS) rats (file number 81-02.04.2021.A111) in accordance with the German Animal Protection Law and approval of the *Landesamt für Natur*, *Umwelt und Klima* of Nordrhein-Westfalen, Recklinghausen, Germany. WT rats were obtained from Janvier Labs and RCS rats (RCS-p+/LavRrrc) were obtained from the Rat Resource and Research Center (RRRC, Columbia, MO, USA) and bred locally. Animals were first deeply anesthetized with carbon dioxide(CO_2_) or a mixture of isoflurane in O_2_ and then decapitated. The experiments were conducted on 3–4 months old female WT rats and on 10-month-old female RCS rats, which are considered to be fully blind. This was tested during the electrophysiological experiments by optical stimulation, which showed no response. Immediately, the eyeballs were enucleated and immersed into fresh Ames’ medium (A1420, Sigma Aldrich, Taufkirchen, Germany) with a pH of 7.4 adjusted with sodium bicarbonate (NaHCO_3_). The medium was constantly oxygenated with carbogen gas containing 95% O_2_ and 5% CO_2_ (The Linde Group, Pullach, Germany). The preparation of the light-adapted retinas was performed as reported earlier [18]. After opening one eyeball along the *ora serrata*, the cornea, lens, and the vitreous body were carefully removed. Then, the same process was performed with the second eye to guarantee constant oxygenation. After that, the posterior eyeballs were cut in halves to isolate two pieces of retina of each eye. For the experiment, each half retina piece was placed on a donut-shaped piece of filter paper with the ganglion cell layer (GCL) facing downwards, while the remaining pieces were kept in oxygenated Ames’ medium. Therefore, a total of 16 retinal samples from four WT rats and 8 retinal samples from two RCS rats were used for this study.

**Figure 3:**
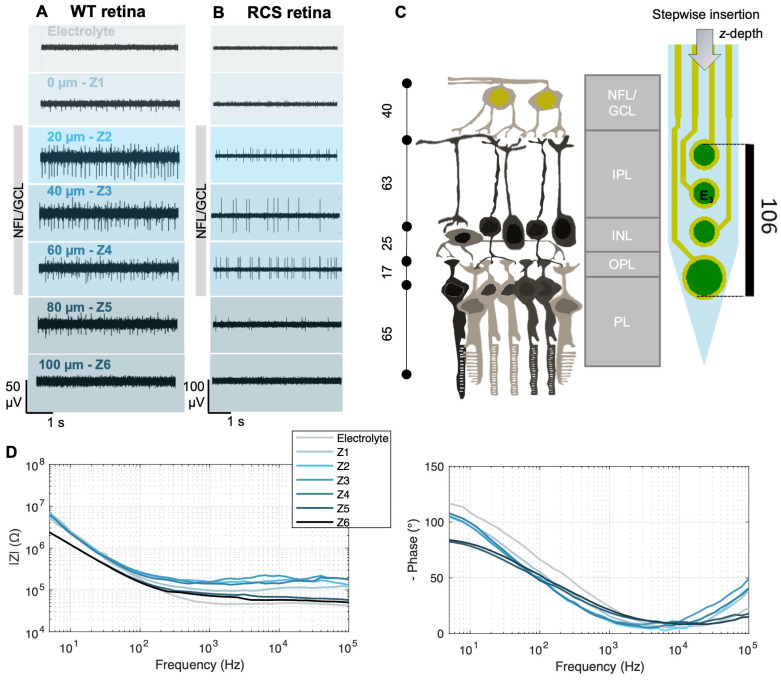
Electrophysiological recordings and impedance measurements at different intraretinal depths. Electrophysiological recordings of WT (**A**) and RCS (**B**) rat retinas at different intraretinal positions *Z_i_*. After an initial insertion step of 100 µm (from the electrolyte to reach Z1), incremental steps of 20 µm were carried out in between *Z_i_*. Data were bandpass-filtered between 100 and 3000 Hz. (**C**) Schematic of retinal layers and their approximate thickness [29,30] in a WT rat retina in comparison with the dimensions of an intraretinal shank. NFL stands for nerve fiber layer, GCL for ganglion cell layer, IPL for inner plexiform layer, INL for inner nuclear layer, IPL for inner plexiform layer, and PL for photoreceptor layer. Dimensions are given in µm. (**D**) Exemplary impedance measurements (left: impedance magnitude; right: impedance phase) from a WT rat retina at the different z-positions Z_i_ and in the electrolyte (Ames’ medium). The data were smoothed using a moving average filter.

With the GCL facing upwards, each explanted piece of WT and RCS rat retina was placed inside a perfusion chamber filled with fresh, oxygenated Ames’ medium. Each piece of retina was fixed with insect pins to hold it in place (Figure 1B,C). This process was repeated for each explanted piece of retina, and electrophysiological and impedance recordings were carried out subsequently (Figure 3).

Each intraretinal probe was first attached to the head stage of the data acquisition system, which was mounted on a micromanipulator. The intraretinal probe was inserted as previously reported [18]. Guided by an optical microscope (VHX, Keyence Deutschland GmbH, Neu-Isenburg, Germany), the intraretinal probe was first positioned near the retinal surface by carefully adjusting its position using the micromanipulator. At the retinal surface (*Z*_0_), no evidence of spiking activity was observed. The intraretinal probe was then inserted into the tissue with incremental steps using an insertion speed of 187.5 µm/s. To overcome tissue dimpling, a preliminary insertion of 100 µm was undertaken to penetrate the GCL and gain access to position *Z*_1_, where small spikes were picked up by the bottom electrode. The probe was then driven deeper into the tissue with incremental steps of 20 µm until the spiking activity of the GCL was captured by the upper electrodes (E i.4 in Figure 1A). A maximum of four insertions with a distance of at least 300 µm were performed per piece. This insertion protocol minimized insertion damage, as demonstrated in previous studies [18].

### 2.4. Electrophysiological Recordings, In Vitro Impedance Measurements, and Signal Processing

Electrophysiological and EIS measurements were carried out inside a Faraday cage using separate systems (see Figure S1). For in vitro tissue recordings, the ME2100-System (Multi Channel Systems MCS GmbH, Reutlingen, Germany) and a 32-channel head stage (ME2100-HS32-M-3m) were used. NeuroNexus adapter (ADPT-NN-16/32) served to connect the head stage to our customized 16-channel PCBs. The McsMatlabDataTools Matlab toolbox version v 1.3.1 [24] and self-written scripts were used to import and offline process HDF5 files created by the ME2100-System. The raw traces were bandpass-filtered with cut-off frequencies of 100 Hz and 3 kHz using a 6th-order zero-phased Butterworth filter to extract spiking activity. Spikes in the bandpass-filtered traces were detected using the UltraMegaSort2000 algorithm [25]. The SNR was calculated by the amplitudes of the detected spikes divided by the root-mean-square (RMS) of the noise, which corresponded to a 10 ms long period of the signal devoid of any spiking activity. At each intraretinal depth, spontaneous activity was recorded after at least ten minutes for two to five minutes to ensure tissue recovery upon intraretinal insertion [19], followed by EIS measurements using the same electrode. The impedance was measured using a portable potentiostat (PalmSens4, PalmSens BV, Houten, Netherlands) in the range of 10 to 10^5^ Hz with 51 measuring points/decade using an AC excitation signal of 10 mV. Electrophysiological recordings were carried out using an Ag/AgCl pellet reference electrode, whereas a two-electrode cell setup was used with a Pt reference/counter electrode for impedance measurements. To avoid ground loops and electrical interference, the acquisition of the MCS data acquisition was turned off, the Ag/AgCl reference electrode used for electrophysiological recordings was disconnected during EIS measurements and the Pt wire, likewise, was disconnected during the electrophysiological recordings. Prior to the application of the PRF method, the impedances traces were smoothed using a moving average filter (see Figure S6).

### 2.5. Resistivity Measurements

Resistivity was derived from EIS measurements using the PRF method [14,15]. First, the frequency at which the impedance of the interface is most resistive is identified, i.e., where the phase *φ_PRF_* is closest to zero degrees (Figure 2A). The magnitude of the impedance at this phase *|Z_PRF_|* was then registered and converted to resistivity as follows.

The cell constant, which is a factor that relates the electrical conductivity of an electrochemical cell with its geometry, was computed using Equation (2):

(2) $$c=\kappa \cdot R_{E}$$

Here, *κ* is the electrolyte conductivity and *R_E_* is the electrolyte resistance. In this case, the electrolyte is the medium in which the retina is immersed, i.e., Ames’ medium (*κ =* 1.546 S/m). To compute *R_E_*, the electrode-tissue/electrolyte interface was characterized by using a modified Randles model (Figure 2B). The model comprised a double-layer capacitance *CE* represented by a constant phase element (CPE), a charge transfer resistance *R_CT_,* a tissue/electrolyte resistance *R_Z_* and a parallel parasitic capacitance *C_P_* (Figure 2B) [6,15]. When the electrode is immersed in the electrolyte but remains outside of the tissue, *R_Z_* equals *R_E_*. The electrical double layer parameters were first derived from the impedance measurements carried out during electrode characterization using 1xPBS as the electrolyte (Figure S2). Then, the impedance fit was carried out in Ames’ right before the start of the measurements. The values of the impedance fit in Ames’ are given in Figure 2C yielding an equivalent capacitance *C_EDL_* of 7.69 pF and an *R_E_* of 46.04 kΩ. The extraction of *R_CT_* was performed directly from the bode plot after fitting the remaining circuit elements, as the fitting procedure exhibited numerical instability and yielded high errors.

During electrode insertion (Section 2.3), impedance measurements were taken at each insertion step (*Z_i_*). Once the electrodes were positioned within the tissue, *R_Z_* reflected the combined contribution of the tissue resistance (*R_T_*) and *R_E_*. Accordingly, the resistance at each intraretinal depth *R_Zi_* was determined using the PRF method. To isolate *R_T_* at each depth, *R_T_* was calculated by subtracting *R_E_* from *R_Zi_*. The corresponding tissue resistivity (*ρ_i_*) was then computed according to Equation (3):

(3) $$\rho_{i}={(R}_{Z_{i}}-R_{E})/c$$

Consequently, resistivity measurements are not affected by different electrode diameters as the values are normalized by the cell constant of each electrode. After multiple measurements within the retina, each resistivity trace was aligned by its peak value (see Figure S3), and the average, standard error mean (SEM) and standard deviation (SD) at each measuring point were computed. Alignment ensured that all measurements started at the same *z*-depth (the retinal surface), as data were collected from multiple electrodes at different retinal depths.

### 2.6. COMSOL Simulations

To analyze the electric field upon current-controlled stimulation, a two-dimensional AC/DC model was employed in COMSOL Multiphysics version 5.6. The retinal thickness and resistivity profile were modelled using the results presented in Section 2.1. Accordingly, a retinal thickness of 240 μm was selected for WT and 120 μm for RCS rat retinas, respectively. An interpolation function was employed to define the resistivity values as a property when the material of the retina was specified. A 25 μm PEDOT/PSS electrode with a conductivity of 101 S/cm [26] was positioned at a depth of 70 µm in the inner retina (INL/IPL), which is presumed to be a good location for retinal stimulation [27]. The stimulation pulse was implemented as a normal current density *J_stim_* according to Equation (4):

(4) $$J_{stim}=I_{stim}/GSA$$

with the geometrical surface area GSA of the electrode. A parameter sweep of the stimulation pulse *I_stim_* was conducted, ranging from 0.5 to 15 μA with a step size of 0.5 μm, to assess the spread of the electric field. For the evaluation of cell activity, a 10 μm long neuronal cell was considered, necessitating an electric field of approximately 3000 V/m for depolarization considering a cross-cellular potential of 30 mV [28]. This represents the minimum electric field, referred to as the electric field threshold (*E_th_*), which is sufficient to activate the tissue [28].

### 2.7. Statistics

After confirming the normality of the data using a Lilliefors test, unpaired *t*-tests with 95% confidence intervals were performed to compare the resistivity values and features extracted from the electrophysiological data (e.g., spike amplitude, root mean square of the noise, and signal-to-noise ratio) between the WT and RCS retinas across retinal depths. To analyze the relationship between the resistivity values and electro-physiological data, cross-correlations and Spearman correlation coefficients were calculated. Subsequently, to account for the considerable standard deviations and limited sample size, the data underwent bootstrapping with 1000 samples to analyze the difference in means between the RCS and WT data. Furthermore, the coefficient of variation (CV) was calculated using Equation (5):

(5) $$CV=\frac{\sigma_{Zi}}{\mu_{Zi}}$$

Here, the ratio of the standard deviation $\sigma_{Zi}\;$to the mean value $\mu_{Zi}$ was computed for each retinal depth *Z_i_* for both, the resistivity data and the electrophysiological metrics. The statistical analysis was performed using MATLAB (MathWorks, Natick, MA, USA) version R2021a, equipped with the Statistics Toolbox. This implementation incorporated a series of functions, including lillietest(), ttest2(), xcorr(), corr(-,‘Type,’ ‘Spearman’), and bootstrp() for Lilliefors test, unpaired *t*-tests, cross-correlations, Spearman coefficients, and bootstrapping, respectively.

## 3. Results and Discussion

### 3.1. Impedance Measurements and Electrophysiological Activity of WT and RCS Rats

Intraretinal probes enabled the recording of electrical activity within the retina. Representative recordings from a single electrode (E3) at various intraretinal depths are shown for both WT (Figure 3A) and RCS (Figure 3B) retinas; recordings from additional electrodes are provided in Figure S4. Following the initial insertion step, electrodes at the tip of the shanks first detected the spiking activity of the retina, indicating proximity to the GCL, where RGCs, the only neurons firing action potentials in the retina, are located. As the probe advanced towards the GCL, the spike amplitudes increased (*Z*_1_–*Z*_2_ in WT, Figure 3A; *Z*_1_–*Z*_3_ in RCS Figure 3B), then decreased when moving away from the GCL (*Z*_3_–*Z*_5_ in WT, Figure 3A; *Z*_4_–*Z*_5_ in RCS Figure 3B) and disappeared when the electrodes approached the inner retina towards the photoreceptor side (*Z*_6_ in WT and RCS, Figure 3A,B).

This depth-dependent shift in spike detection, from lower to upper electrodes as the shank is fully embedded in the retina, together with the probe dimensions (Figure 3C), further provided a framework for estimating electrode placement within the retina. Accordingly, the presence of high-amplitude spiking activity when electrodes were positioned at a depth of 20 to 60 µm (Z_2_–Z_4_) in both WT and in RCS retinas suggests that the electrodes were located within the nerve fiber layer (NFL) and GCL, layers that are estimated to be 40 µm thick [29,30]. At *Z*_1_ in WT and *Z*_1_*–Z*_2_ in RCS, low-amplitude spikes were recorded, indicating positioning at the retinal surface, close to the NFL/GCL. The electrodes appear to be fully positioned within these layers only when spike amplitudes reached their peak, at *Z*_2_ for WT and *Z*_3_ for RCS. Based on the spike amplitudes and the insertion increments following *Z*_2_, the depth of electrode E3 at *Z*_3_, shown in Figure 3A, is estimated to be 40 µm (Figure 3A,B and Figure S4).

Consequently, only the bottom electrode of the shanks could reach the photoreceptor layer within WT retinas (Figure S4). These observations are consistent with previous studies using intraretinal probes [18,19,20]. It is important to note though that, given the viscoelastic properties of the retina, accurate initial placement of the intraretinal probe is critical for penetrating the GCL. If the probe is not correctly positioned at the retinal surface during the first insertion step, additional insertion steps are required to penetrate the GCL due to tissue dimpling [18,19,20]. Nonetheless, positioning at the GCL can be identified by its characteristic recordings with high spike amplitudes, which allow for determination of intraretinal depth according to insertion increments and probe dimensions (Figure 3C).

In the RCS rat, in conjunction with the spiking activity, neuronal oscillations in the low-frequency range were measured (Supplementary Figure S5) [31]. These oscillations have been observed similarly in other animal models of degenerated retinas (*rd1* and *rd10* mice) and were the subject of comprehensive studies in other investigations, due to their influence on the efficiency of electrical stimulation protocols [31,32,33]. The ability to detect spikes is contingent not only on penetration depth, but also on the horizontal (*x* − *y*) location of the penetrating shank, given that cell density varies from central to peripheral retina [34]. Furthermore, the presence of blood vessels may impede signal detection due to an absence of cells in those areas. Consequently, the spike amplitudes detected by each electrode and shank during multiple insertions exhibit variability.

Guided by the electrical recordings, we conducted impedance measurements at different intraretinal depths, as illustrated in Figure 3D for a WT rat retina (see Supplementary Figure S6 for an RCS rat retina). While the bottom electrode remained in the electrolyte, impedance showed minimal variation. However, upon approaching the retinal surface (position *Z*_1_), the electrode began to partially penetrate the NFL/GCL (Figure 3A), at which point a noticeable change in the impedance was typically observed. Compared to measurements in the electrolyte, the impedance magnitude |*Z*_1_| was consistently higher across most frequencies, while the phase trace exhibited a slight deviation in its maxima toward zero. This trend persisted with increasing intraretinal depth until reaching a specific point, designated as *Z*_5_ in Figure 3A, where the impedance magnitude began to decline. Typically, the decline in impedance (*Z*_5_) began shortly after the decline in amplitude (*Z*_3_) of the spiking activity. The trend of the impedance measurements is consistent with previous measurements carried out in explanted mouse retinas [6].

### 3.2. From Impedance Measurements to Resistivity Profiles Using the PRF Method

**Figure 4:**
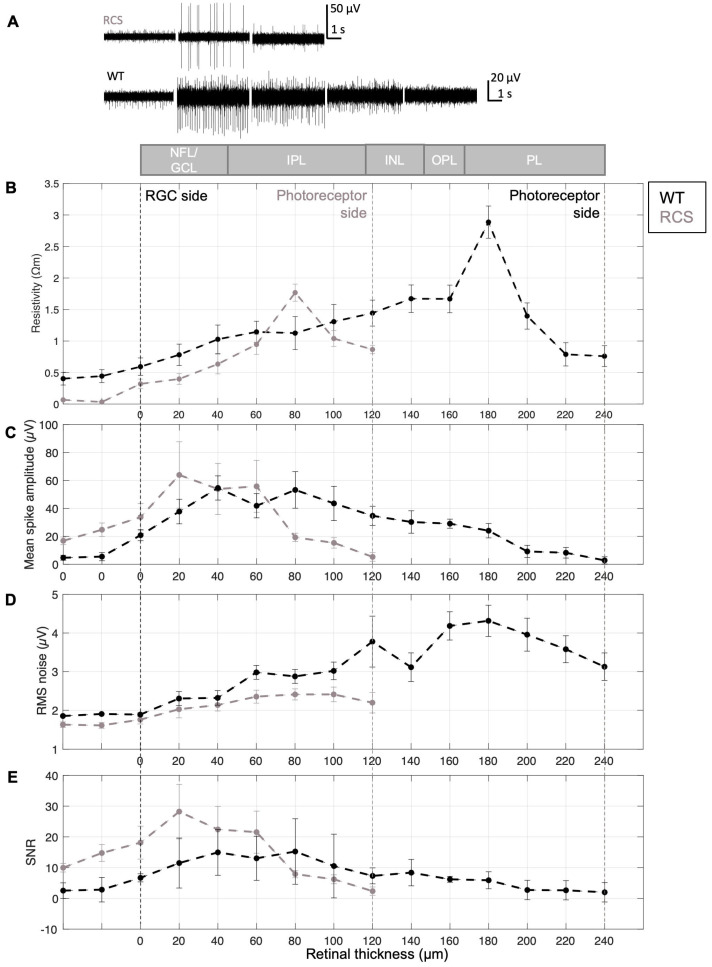
Resistivity profile and electrical recording quality. (**A**) Resistivity profile (*N* = 12), (**B**) mean spike amplitude, (**C**) representative electrophysiological signals, (**D**) RMS noise, and (**E**) signal-to-noise ratio (SNR) across retinal layers in WT and RCS rats. A schematic at the top illustrates the expected retinal layers across depths, including the nerve fiber layer (NFL), ganglion cell layer (GCL), inner plexiform layer (IPL), inner nuclear layer (INL), inner plexiform layer (IPL), and photoreceptor layer (PL). The examples displayed in (**C**) were recorded at the retinal surface (0 µm), and at depths of 40, 80, 120 and 160 µm. Panels (**B**–**E**) are based on *N* = 6 WT/7 RCS. Data are presented as mean ± SEM (plots with mean ± SD are presented in Figure S7).

When the electrode traverses the retina, *R_Z_* represents the sum of *R_T_* and the *R_E_*_._ In this context, *R_T_* is assumed to reflect the resistance of retinal neurons as well as the extracellular fluid present between them. Although noise was present in the EIS data due to experimental factors, such as bubbling of the retina with carbogen to preserve tissue vitality, adsorption of organic and ionic species on the electrode surfaces, and the influence of stray capacitances from the experimental setup at higher frequencies (evidenced by a drop in impedance magnitude above 10^4^ Hz; Figure S6), the observed impedance changes across *Z*-depths are indicative of the depth-dependent resistivity profile of the retina (Figure 3D). In Figure 4A, the RGC side is positioned on the left and is aligned for both groups, while the photoreceptor side is on the right and differs between WT and RCS rats. The change in resistivity values upon retinal penetration marks the probe’s entry, with boundaries defined by a 10% deviation from the initial baseline [6]. Within these boundaries, retinal thickness was determined, yielding values of 240 µm for WT and 120 µm for RCS rats, which is consistent with previously reported values for healthy and fully blind RCS retinas, respectively [30].

As revealed by Figure 4B, the resistivity profile displays a non-monotonic trend, with resistivity increasing from the retinal surface toward deeper layers, rising from lower values in the NFL/GCL to higher values in the inner retina. This increase is consistent with expectations, as neuronal density is known to rise in deeper retinal layers. At the RGC side, the resistivity was 0.32 ± 0.27 Ωm for RCS and 0.59 ± 0.48 Ωm for WT retinas. Resistivity increased with electrode insertion depth, reaching a peak of 1.76 ± 0.49 Ωm (*N* = 12) in RCS at 80 µm and 2.89 ± 0.88 Ωm (*N* = 12) in WT at 180 µm, corresponding to approximately 66% and 75% of the total retinal thickness, respectively. Beyond these depths, resistivity decreased. Reported literature places the resistivity peak within 65–80% of the retinal thickness in rodents [6,7], supporting the interpretation that the increase is driven by higher neuronal density in these regions.

Accordingly, a statistically significant difference was identified when comparing the peak values of the resistivity of WT and RCS rats (*p* = 0.004, using an unpaired *t*-test with a confidence interval of 95%, *N* = 12), as evidenced by the spatial separation of the peaks. Beyond this peak, resistivity decreased towards the photoreceptor side. However, the measured resistivity did not return to baseline, likely due to the use of a soft insulating PDMS substrate beneath the tissue and the fact the intraretinal probes (225 µm) were shorter than the thickness of WT retinas (240 µm).

To validate the PRF method, resistivity peaks were calculated from impedance fits. The cell constant was subsequently calculated from the fitted *R_z_*, enabling the determination of tissue resistance and subsequent resistivity calculation. This approach yielded a peak resistivity of 2.81 ± 1.00 (*N* = 10 measurements) for WT rat retinas, which is analogous to the value obtained with PRF (2.89 ± 0.88, *N* = 10 measurements), thereby supporting the validity of the method.

### 3.3. Relating Electrophysiological Data to the Resistivity Profile

From the electrophysiological data, signal quality patterns were identified that can be attributed to the position of the electrodes within the retina and the resistivity at each of those *z*-depths. The voltage measured by an extracellular electrode is inversely proportional to the distance between the electrode and the current source, in this case RGCs, and directly proportional to the local resistivity of the extracellular medium, as supported by the point-source equation and volume conductor models [35]. Thus, both the spatial arrangement of the electrodes and their distance from the neuronal source significantly influence the quality of the recorded signal. In addition, the voltage measured at the electrode is affected by the electrical properties of the electrode–tissue interface, particularly the effective electrode impedance and input impedance of the recording system [23].

Consequently, the amplitude of spiking activity increased to its peak as the probe reached the GCL, as shown in Figure 3A and Figure 4B,C, and remained relatively constant within the GCL (at a retinal depth of around 20 to 60 or 80 µm, Figure 4B for RCS and WT retinas, respectively). At the GCL, both resistivity and distance between RGCs and electrodes are minimal, resulting in larger potential recordings. After the GCL, the amplitude of the spiking activity decreased, for WT as well as RCS retinas, until the spikes are not distinguishable from the background noise in deep retinal layers. Given the variability in the insertion process and the location- and distance-dependent nature of signal amplitudes, high standard deviations of spiking amplitudes are expected (Figures S7 and S9). A comparison of spiking amplitudes in RCS and WT retinas shows slightly higher amplitudes in RCS retinas. This increase can be attributed to a lower-resistivity extracellular medium in RCS retinas, which facilitates the current flow from neuronal sources, particularly when these sources, such as RGCs, are in close proximity to the recording electrodes. Besides, the thinner RCS retina comprises a more challenging retina preparation and increases susceptibility to damage cells at both surfaces of the retina (RGC and degenerated photoreceptor side), which can further hinder electrophysiological recordings and thereby lead to a higher standard deviation of the presented data. Although spike amplitude differences were minimal, unpaired *t*-tests revealed no statistically significant difference between RCS and WT data. Similarly, the comparison of bootstrapped means for the electrophysiological profiles of WT and RCS showed no significant difference, as the confidence interval of the mean difference included zero (Figure S10).

A comparison of retinal layer resistivity and spike amplitude reveals an inverse relationship: as resistivity increases, amplitude tends to decrease. However, this decrease in amplitude is not solely attributable to higher resistivity; it is also influenced by the increasing distance between the recording electrodes and the neuronal sources. Notably, the highest amplitudes are observed in the GCL, where RGCs are located. In contrast, the outer retinal layers, which exhibit the highest resistivity, primarily contain non-spiking neurons, such as intermediate neurons and photoreceptors. Cross-correlation analysis of the resistivity and spike amplitude reveals a shifted relationship, reflecting the spatial separation of spike amplitudes and resistivity peaks across different retinal layers (Figure S8A,B).

In turn, noise increased during intraretinal recordings in deeper retinal layers (Figure 4D), for both WT and RCS rats. The noise of electrophysiological recordings is influenced by external and intrinsic factors. External noise sources include interference from power lines, light sources, instrumentation noise from recording hardware (e.g., amplifiers, analog-to-digital converters, and connecting cables), as well as biological noise arising from the activity of distant neurons. On the other hand, thermal noise is the primary source of intrinsic noise and is strongly influenced by the impedance of the microelectrodes. For the microelectrodes utilized in this study, the impedance at 1 kHz was as low as 33.8 ± 1.9 kΩ for small electrodes with a diameter of 15 µm (*N* = 14) and 16.9 ± 0.1 kΩ for large electrodes with a diameter of 25 µm (*N* = 3), respectively (Figure S2C). The thermal noise of the microelectrodes in saline was 1.05 µV for small and 0.8 µV for large electrodes, respectively.

However, it is important to note that noise in electrophysiological recordings depends on the components of the neural interface at all levels, not just the electrode itself. As previously stated, the recorded voltage is directly proportional to the local resistivity of the extracellular medium. While external noise sources remain unchanged during measurements at varying retinal depths, changes at the electrode–tissue interface, resulting from resistivity variations across retinal layers, are the predominant contributors to noise levels during electrophysiological recordings. Thus, RMS of the noise increases with retinal depth, mirroring the concurrent increase in resistivity (Figure 4B,D). This relationship is supported by Spearman’s rank correlation, which revealed strong positive correlations between resistivity and RMS noise, 0.80 for WT and 0.98 for RCS (see Figure S8C,D). In WT retinas, RMS noise peaks at a depth of 180 µm before declining, closely following the resistivity profile. In RCS retinas, the peak occurs at 80 µm and similarly decreases thereafter, also reflecting the resistivity trend. These observations support the assumption that noise levels are influenced by the electrode–tissue interface [36].

Furthermore, the SNR, defined as the ratio of spiking amplitude to RMS noise, peaks in the GCL and declines as RMS noise increases and spiking amplitude decreases in both WT and RCS rat retinas. The high SNR in the GCL corresponds to its low resistivity, which facilitates the detection of high-amplitude spiking activity with minimal noise. In deeper retinal layers, increasing resistivity and the greater distance between electrodes and spiking RGCs reduce signal amplitude and elevate noise levels, leading to a lower SNR. A discrepancy in the mean values of the WT and RCS datasets (see Figure S10) suggests underlying differences in the data distributions, particularly within the NFL/GCL (initial 20 µm depths), where RCS recordings show markedly reduced noise RMS and increased SNR compared to WT (Figure S10). This observation is consistent with lower tissue resistivity, which enhances signal amplitudes and reduces noise levels (Figure 4C,D). However, this trend reverses in deeper retinal layers, especially beyond the point of peak resistivity observed in RCS rats, observed at an approximate retinal depth of 80 µm. At this depth, SNR in RCS rats decreases relative to WT, likely due to reduced spike amplitudes and elevated noise. These findings highlight the relationship between electrophysiological recording quality and resistivity across retinal depths.

Pronounced standard deviations were observed in the data (see Figure S7). The CV for resistivity and electrophysiological metrics was generally elevated at the initial stage (electrolyte) and at the final stage (deep retinal layers), likely due to reduced sample sizes near measurement boundaries. The latter is attributed to the necessity of an alignment step, a prerequisite for the analysis of the data that results in a decrease in sample size at the limits of the measurement. However, within retinal depths up to 100 µm in RCS and 220 µm in WT rat retinas, CV values remained below 40% across all measurement points. Resistivity and RMS noise showed lower variability (CV between 6 and 32%), whereas amplitude and the directly related SNR metrics exhibited higher variability (between 15 and 39%).

High variability in spiking amplitude is expected, as it depends on electrode insertion depth and is contingent on the precise spatial relationship between the spiking cell and electrodes, which is subject to variation from insertion to insertion. In addition, the relatively high standard deviation may, in general, result from several factors. First, external influences, such as instrumentation noise (e.g., stray capacitances from cables), could affect impedance measurements. Second, slight variations in retinal layer composition across different *x*–*y* positions, from the center to the periphery, may contribute to the observed variation [34]. Third, measurement variability can arise from differences in estimating the initial retinal surface position before probe insertion. This variability is evident in electrophysiological recordings, as discussed in Section 3.1 where, in some cases, proximity to RGCs is reached already at *Z*_2_ (Figure 3A, WT), while in others it occurs at *Z*_3_ (Figure 3B, RCS). Despite this variability, the intraretinal resistivity profiles measured in both WT and RCS rat retinas were consistent with previously reported findings in mouse retinas [6]. Overall, CV values indicate similar variability levels in both animal models with elevated but acceptable variation.

### 3.4. Simulations of Current Spread Using the Measured Resistivity Profile

The resistivity profile should also be considered in the context of retinal stimulation, whether applied intraretinally or at the inner (photoreceptor side) or outer (GCL side) surface boundaries. Specifically, the resistivity profile exerts a significant influence on the electric field spread that occurs due to the applied current density when electrically stimulating the retinal cells. As such, it is imperative to take this profile into account during simulations in order to accurately estimate activation patterns.

**Figure 5:**
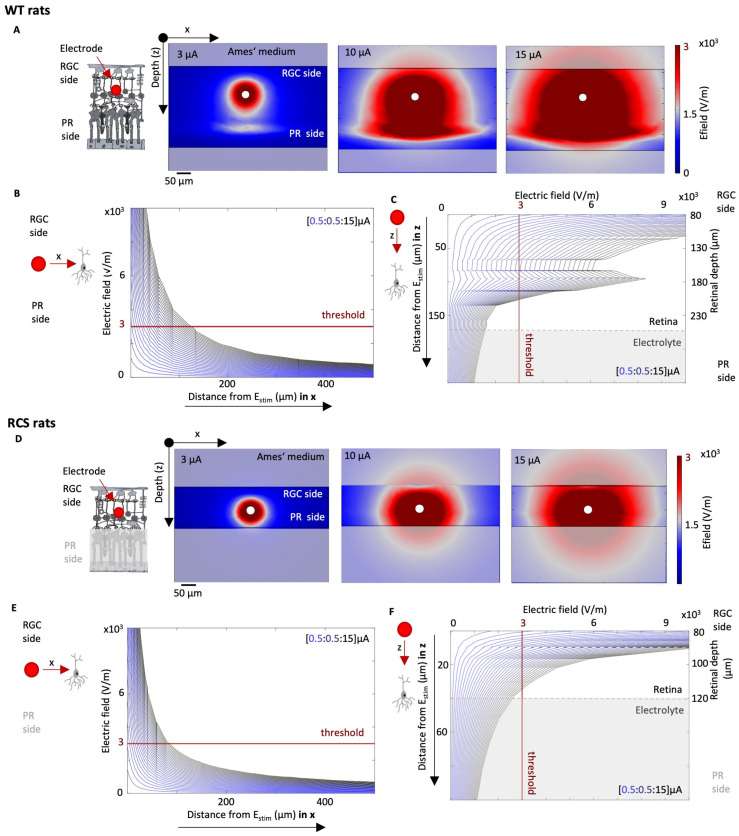
Simulation of electrical stimulation of WT and RCS retina. The electric field spread is evaluated for a range of stimulation pulses *I_stim_* with varying currents and a pulse period of 0.5 ms. The norm of the electric field for a *I_stim_* of 3, 10, and 15 μA is shown in A_i_ and B_i_ for WT (**A**) and RCS (**D**) rat retinas, respectively. The electric field norm depending on the distance to the stimulation electrode in *x* and *z* direction is shown for WT (**B**,**C**) and RCS (**E**,**F**) rat retinas, respectively, while varying *I_stim_* from 0.5 to 15 μA in steps of 0.5 μm.

FEM simulations of the electric field spread upon intraretinal stimulation considering the resistivity profile of WT and RCS rat retinas are presented in Figure 5. While the human retina differs to a large extent between the center and periphery, in the rodent’s retina the effect is not as strongly pronounced [34]. Therefore, variations of resistivity in *x*–*y* directions were neglected. Thus, when the stimulating current (*I_stim_*) is increased, the strength of the electric field also rises. This is demonstrated in Figure 5A,D, which show the effects of *I_stim_* equal to 3, 10, and 15 μA in both WT and RCS rat retinas. The dark red area represents the activation threshold *E_th_* of 3000 V/m, indicating the portion of the retina where, in the event of a cell being present, activation would occur [28].

The distribution of the electric field spread depends on the local resistivity profile, for both WT and RCS rat, given that the electric field E is the product of the resistivity ρ of the surrounding tissue and the current density *J* (E=ρ·J). Figure 5B,C illustrates the evaluation of the electric field in a WT rat, according to the distance of the electrode in the *x* and *z* directions, respectively, with the *z*-direction corresponding to the different retinal layers. In the *x*–*y* direction, the electric field decays exponentially with increasing distance to the stimulation electrode (Figure 5B). In the *z*-direction, the resistivity profile of the retinal layers shapes the electric field distribution, resulting in pronounced peaks at a retinal depth of 180 µm, corresponding to the resistivity peak observed in the WT retina (Figure 4B and Figure 5C). Figure 5E,F presents the same data for RCS rat retinas. In the *x* direction, *E_th_* in RCS rats is reached with higher currents in comparison to WT rats. For instance, to stimulate a cell which is located 50 μm away from the stimulating electrode in the horizontal plane (here in the *x*-direction), an *I_stim_* of 7.5 μA is sufficient for a WT retina, whereas 9 μA is required for an RCS retina. This represents, in turn, an *I_stim_* increase of 21%. At a closer distance of only 10 µm, 2 µA would be sufficient for both WT and RCS rats. Similar observations are evident in the *z*-direction towards the deeper layers of the retina. For instance, when indirect stimulation of the ganglion cells is desired, e.g., of the bipolar cells which are in the INL layer, the current required is 9.5 μA at a distance of 50 μm in RCS rats and only 6 μA in WT rats, an increase of 58%. At a smaller distance of 10 µm in the *z*-direction, the required *I_stim_* for WT and RCS rats is 2 µA. These findings highlight how the resistivity profile largely influences the spread of the electric field upon electric stimulation and thereby the cell activation, leading to larger thresholds in RCS rats in comparison to WT rats.

## 4. Conclusions and Outlook

This study measured intraretinal resistivity across layers in both, WT and diseased RCS rat retinas, revealing a resistivity profile consistent with previously reported findings for healthy and diseased mouse retinas [6]. The alterations in the pathological oscillations throughout different ages of RCS rats have been documented [31]. Consequently, it is plausible that resistivity is also subject to divergence. Although recordings were conducted on 10-month-old RCS rat retinas, additional measurements at different ages are needed to gain deeper insights into the timing and progression of resistivity changes upon retinal degeneration. In addition to implementing measures to mitigate external noise, such as enhancing the shielding of the measurement system, it would be advantageous to assess alternative methods for extracting the resistance. This is because external noise can compromise the precision of the PRF method during impedance measurements, as evidenced by the relatively high standard deviations introduced by experimental factors. It is hypothesized that the least-squares resistance calculation is more robust to noise. However, the near-ideal capacitance (close to 1, as illustrated in Figure 3B) exhibited by the impedance fit of the electrodes in this study is a critical factor in the accuracy of the PRF method. Consequently, only minor discrepancies from the “true resistance” are anticipated [15]. Thus, the PRF approach was selected in this study due its advantageous rapid measurement and calculation speed, which, in principle, facilitates its application in the context of retinal implant surgery in living subjects.

We demonstrated that multisite penetrating intraretinal probes are a useful tool for analyzing resistivity traces alongside electrophysiological measurements, which is a valuable approach for determining the optimal retinal depth for electrical stimulation. While further histological analysis will be necessary to confirm the precise placement of electrodes within the retina, their intraretinal location can be inferred from resistivity measurements and electrophysiological signals. For instance, estimating the RMS of noise or identifying the absence of spiking activity from retinal ganglion cells at certain depth can provide indirect evidence of electrode positioning. In principle, this approach could be implemented as a closed-loop system by selecting the stimulating electrode based on the measured resistivity and the desired stimulation depth for stimulation, whether the GCL or the inner retina.

Furthermore, electrical stimulation simulations demonstrated that the impact of resistivity changes in the degenerated retina is a critical factor to be considered when developing a protocol for electrically stimulating retinal cells, e.g., implemented in a retinal prosthesis. While simulations were conducted with intraretinal electrodes, retinal resistivity changes across layers also affect the efficiency of epiretinal and subretinal implants, as the spread of the electric field is further influenced by the distance between the stimulating electrodes and the retina. This underscores the need for a bidirectional communication approach in retinal prostheses [20], allowing for the assessment of the diseased retinal network and the dynamic adjustment of electrical stimulation parameters. By incorporating feedback from the cells and accounting for the electrical properties of the retina, this approach could adapt to the progression of degeneration, which varies over time in affected degenerative retinal diseases. During chronic in vivo implantation of retinal implants, electrode impedance is often affected by foreign body reactions, which can result in glial scar formation and device encapsulation [37]. Therefore, applying this methodology in chronic in vivo conditions could also provide valuable insights into the efficiency of electrical stimulation under varying electrode impedance.

## Data Availability

The raw data supporting the conclusions of this article will be made available by the authors on request.

## Supplementary Materials

The following supporting information can be downloaded at: https://www.mdpi.com/article/10.3390/s25123765/s1. Figure S1: Overview of experimental setup; Figure S2: Characterization of the electrode impedance using a Randle cell model; Figure S3. Alignment of resistivity profiles; Figure S4. Intraretinal recordings using flexible penetrating multisite intraretinal probes; Figure S5. Comparison of raw, bandpass filtered, and lowpass filtered electrophysiological signals of RCS and WT rat retinas; Figure S6. Raw and smoothed impedance data at different intraretinal depths; Figure S7. Resistivity profile and recording quality metrics of WT and RCS rat retinas; Figure S8. Relationship of resistivity and recording quality metrics; Figure S9: Coefficient of variation (CV) for WT (black) and RCS (grey) rats of the resistivity measurements and features from the electrophysiological data after bootstrapping the data with 1000 samples; Figure S10: Differences of the means after bootstrapping the WT and RCS data with 1000 samples.
