# Supplementary material for: Intraretinal Electrophysiology and Resistivity Profiles of WT and RCS Rat Retina
Source: Sensors (Basel). 2025 Jun 16;25(12):3765. doi: 10.3390/s25123765 (PMC12197074; doi:10.3390/s25123765)
Supplement: Supplementary file 1 [file sensors-25-03765-s001.zip › sensors-3625793-supplementary.pdf]

Supplementary Material

Intraretinal Electrophysiology and Resistivity Profiles of WT and RCS Rat Retina

Marie Jung[1,2], Antje Willuweit[3], and Viviana Rincón Montes[1*]

[1]Institute of Biological Information Processing (IBI-3), Bioelectronics, Forschungszentrum Jülich, Jülich, Germany

[2]RWTH Aachen University, Aachen, Germany

[3]Institute of Neuroscience and Medicine (INM-4), Medical Imaging Physics, Forschungszentrum Jülich, Leo-Brandt-Str., D-52425 Jülich, Germany

[*]Corresponding author: v.rincon.montes@fz-juelich.de

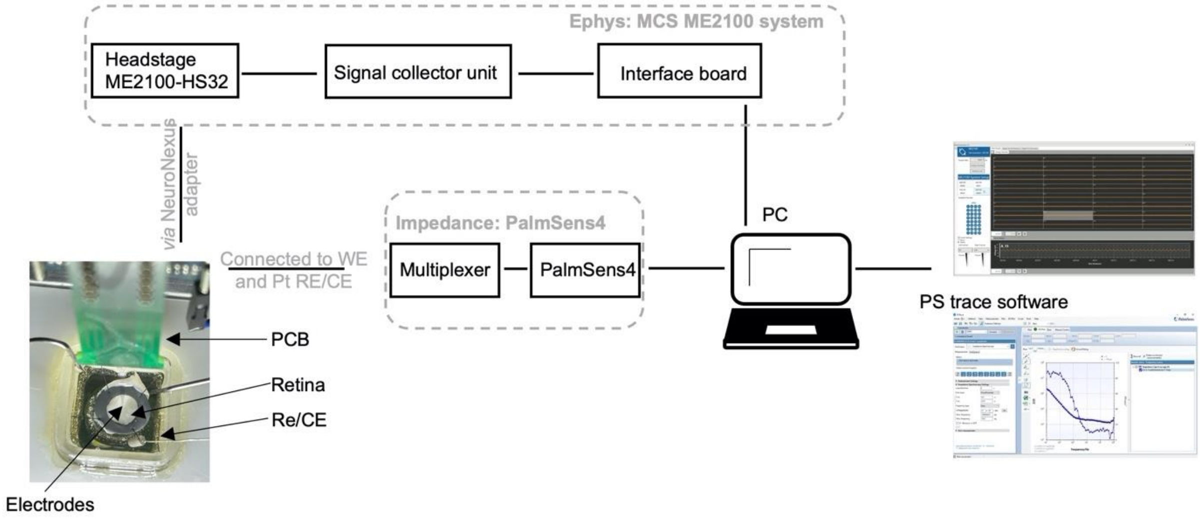

Figure S1. Overview of experimental setup. For the measurement of electrophysiological data, the ME2100 system by Multichannel systems was used. It consisted of a headstage, signal collector unit, and interface unit connected to a PC. The Multichannel Experimenter Software was used to collect and save the data. Impedance was measured with the PalmSens4, which was connected to the same PC. The PS trace software was used to collect and save the impedance data.

A

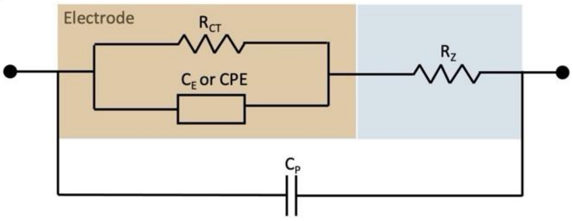

B

Parameter	Value
R_Z	22.76 ± 1.19 kΩ
C_{PE}	7.72 ± 0.02 $nF^{(a-1)}$
a	0.96 ± 0.003
R_{CT}	21.4 ± 0.8 MΩ
C_P	32.3 ± 2.8 pF

C

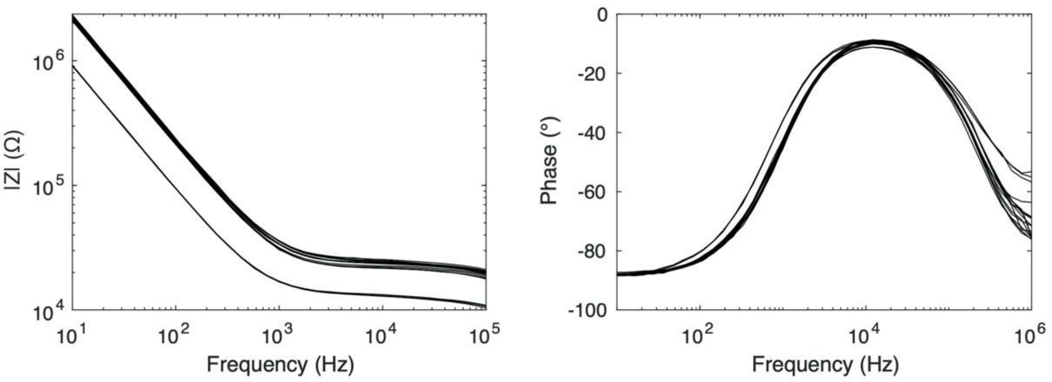

Figure S2: Characterization of the electrode impedance using a Randle cell model. The model comprised a double-layer capacitance *CE* represented by a constant phase element (CPE), a charge transfer resistance R_{CT}, a parasitic capacitance C_P and a tissue/electrolyte resistance R_Z (A) performed with electrodes with a diameter of 15 µm. The fitted values are presented in (B). The goodness of the fit was 0.0872. The raw data of the impedance is presented in (C). The lower impedance values correspond to electrodes with a diameter of 25 µm ($N = 3$), the higher impedances correspond to electrodes with a diameter of 15 µm ($N = 14$).

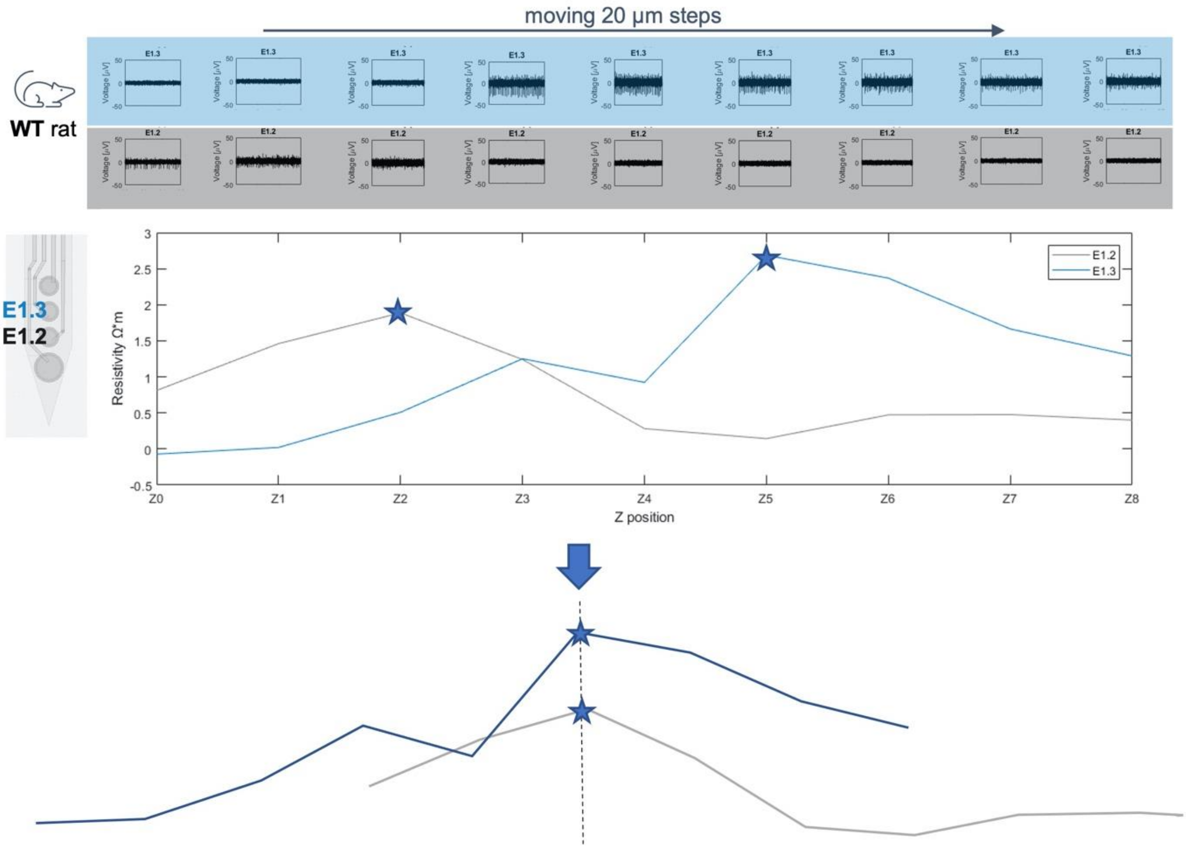

Figure S3. Alignment of resistivity profiles. Electrophysiology and resistivity measurements were conducted using different electrodes within the same shank at varying intraretinal depths. The values were obtained using different electrodes and during several insertions. To facilitate a meaningful comparison of the measurements, the resistivity values were aligned with their respective peaks to determine the average values at each *z*-location. The features of the electrophysiological data were then aligned accordingly.

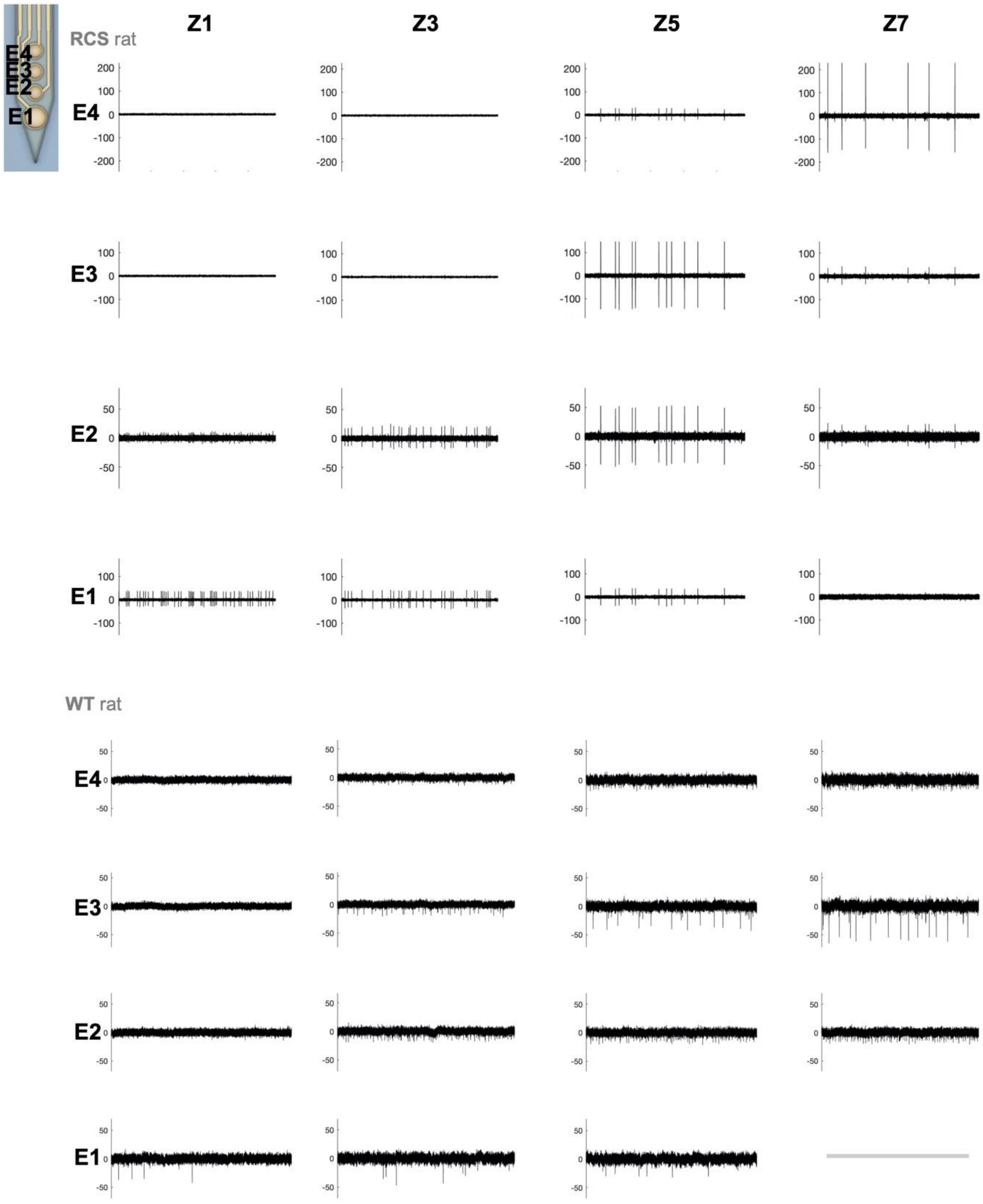

Figure S4. Intraretinal recordings using flexible penetrating multisite intraretinal probes. The data is displayed at Z_1, Z_3, Z_5 and Z_7 of an exemplary recording comprising four electrodes contained on a single shank. The first electrodes (E1) are closest to the ganglion cell layer (GCL) and thus capture spiking activity first. The other electrodes pick up activity as soon as they are near retinal ganglion cells (RGCs). The insertion step size employed was 20 µm. E1 in the WT recording broke at Z_7 (marked with a grey line).

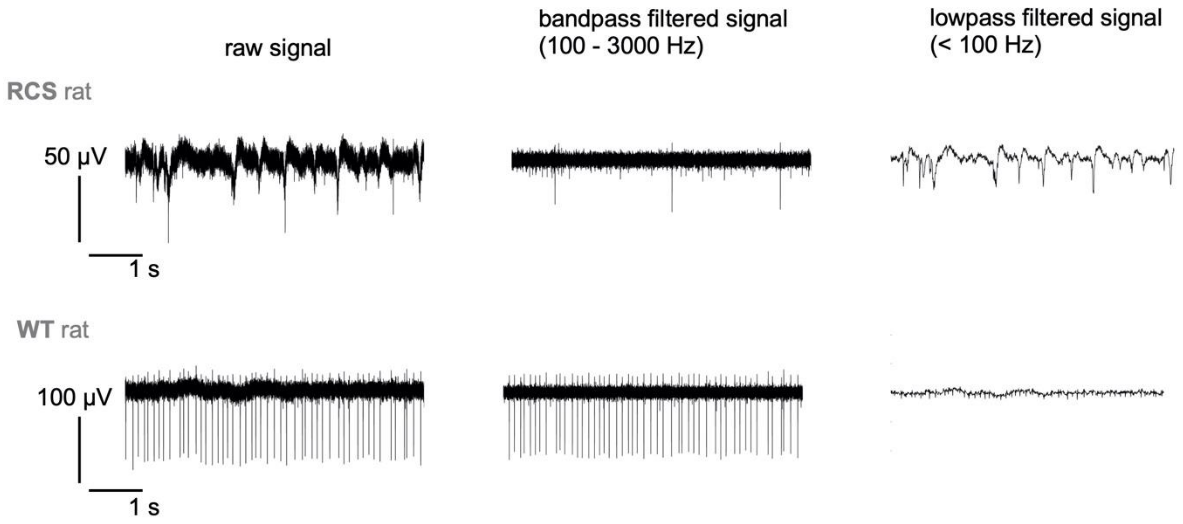

Figure S5. Comparison of raw, bandpass filtered, and lowpass filtered electrophysiological signals of RCS and WT rat retinas.

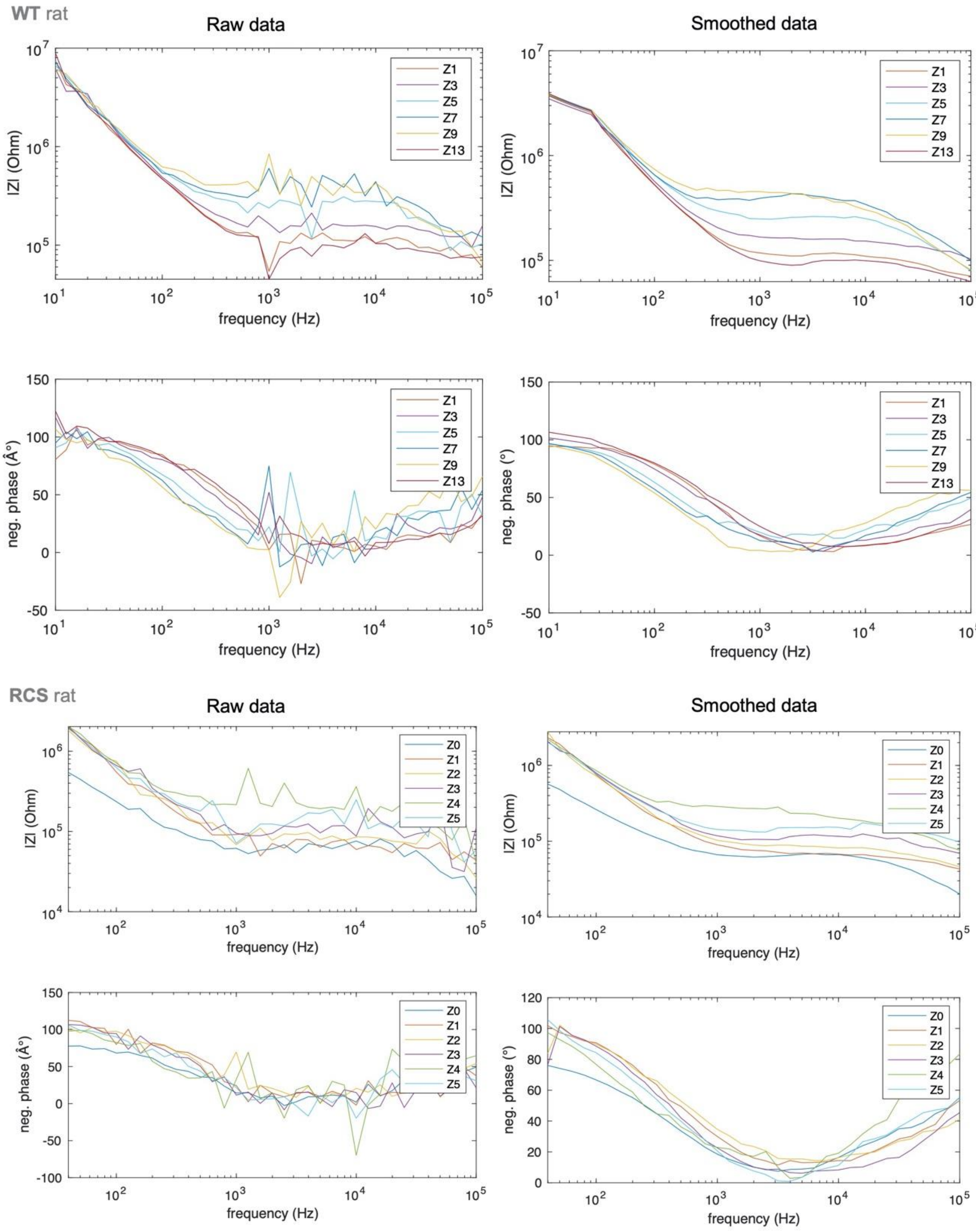

Figure S6. Raw and smoothed impedance data at different intraretinal depths. The measurements were taken on WT and RCS rat retina at the different *z*-positions Z_i and in the electrolyte (Ames' medium, Z_0). The data was smoothed using a moving average filter.

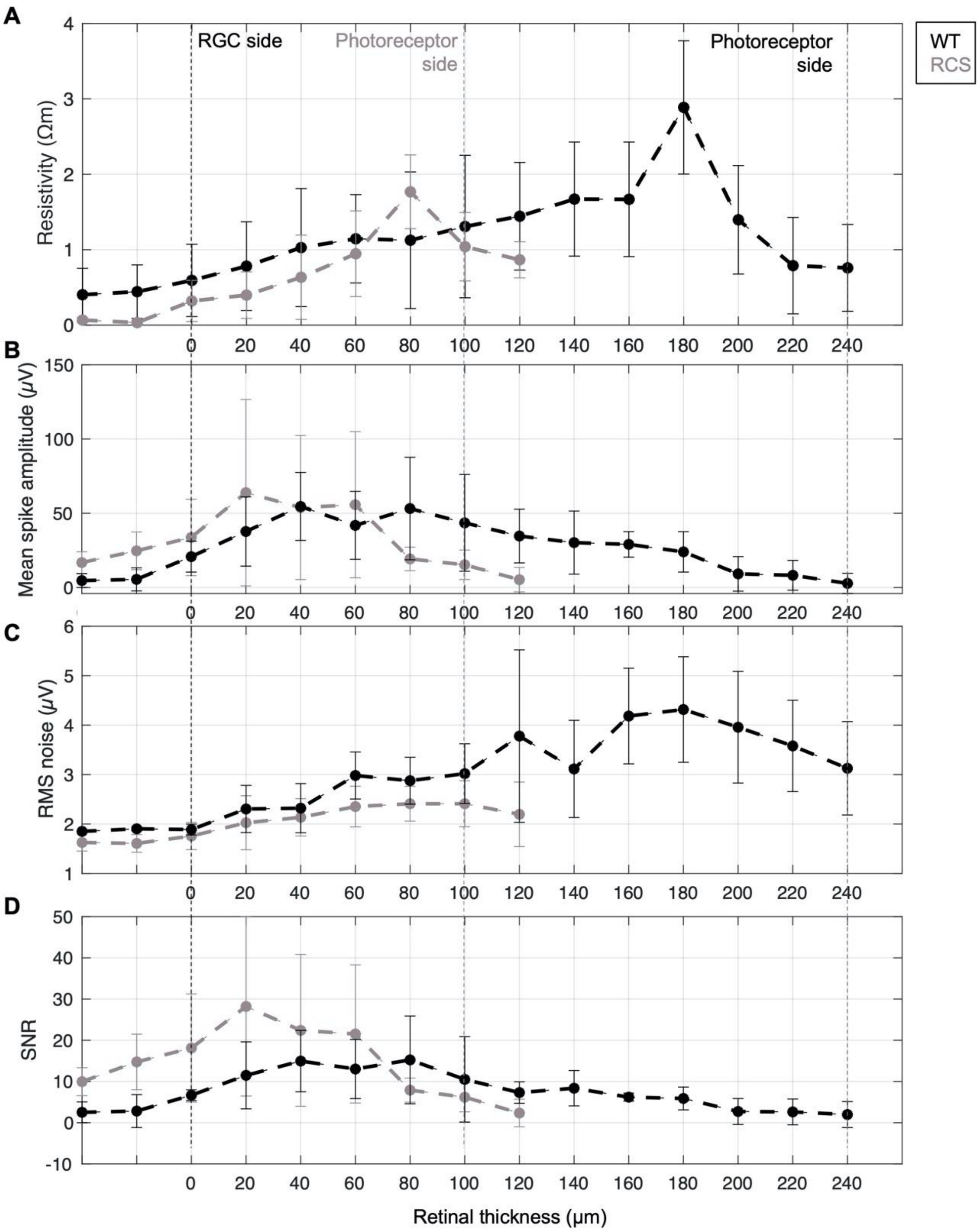

Figure S7. **Resistivity profile and recording quality metrics of WT and RCS rat retinas.** The data shows the mean ± standard deviation of the resistivity (N = 12 WT /13 RCS measurements and the spiking amplitude (N = 6 WT / 7 RCS).

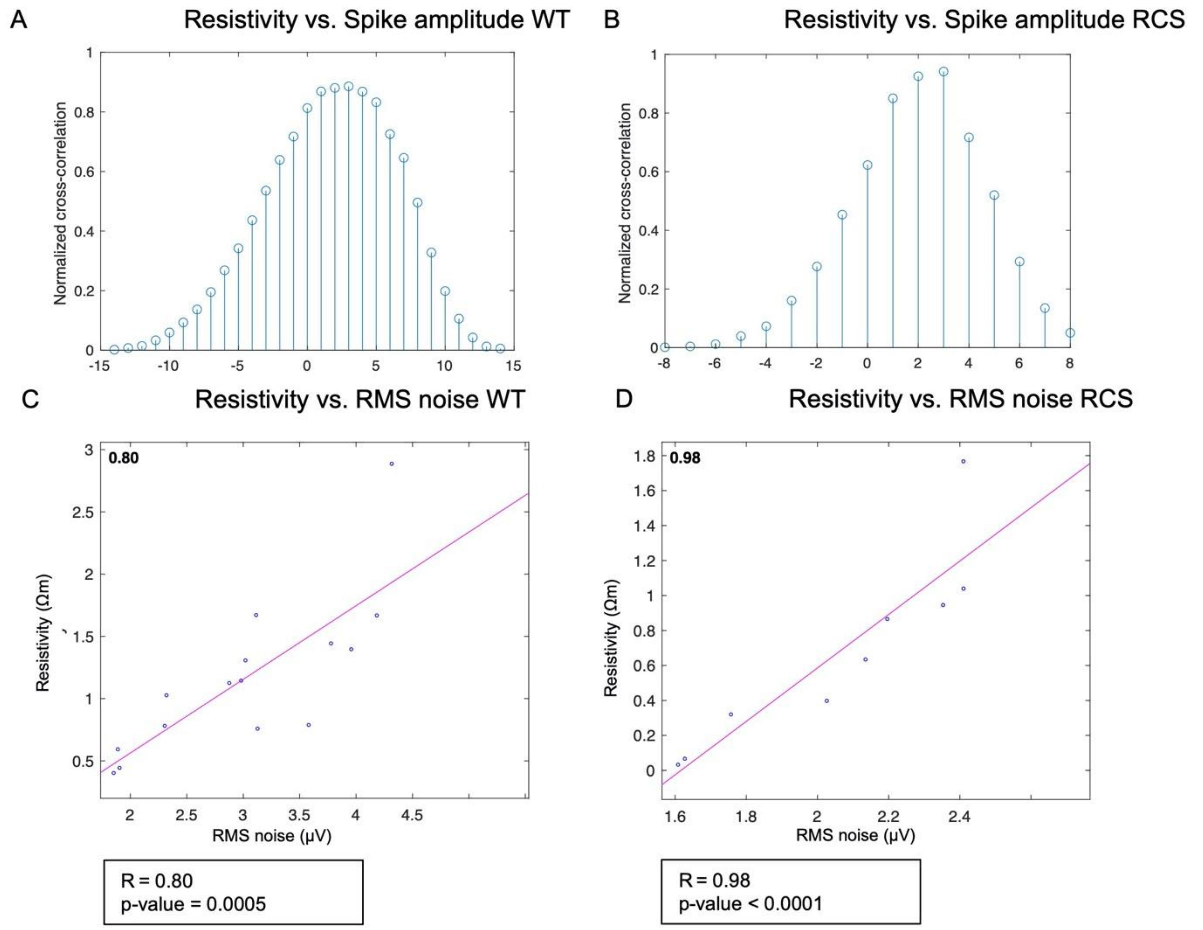

Figure S8. Relationship of resistivity and recording quality metrics. Correlation of the resistivity vs. the spiking amplitude for WT (A) and RCS (B) rat retinas at different intraretinal depths. Spearman correlation analysis of resistivity vs. RMS noise for WT (C) and RCS(D) rat retinas.

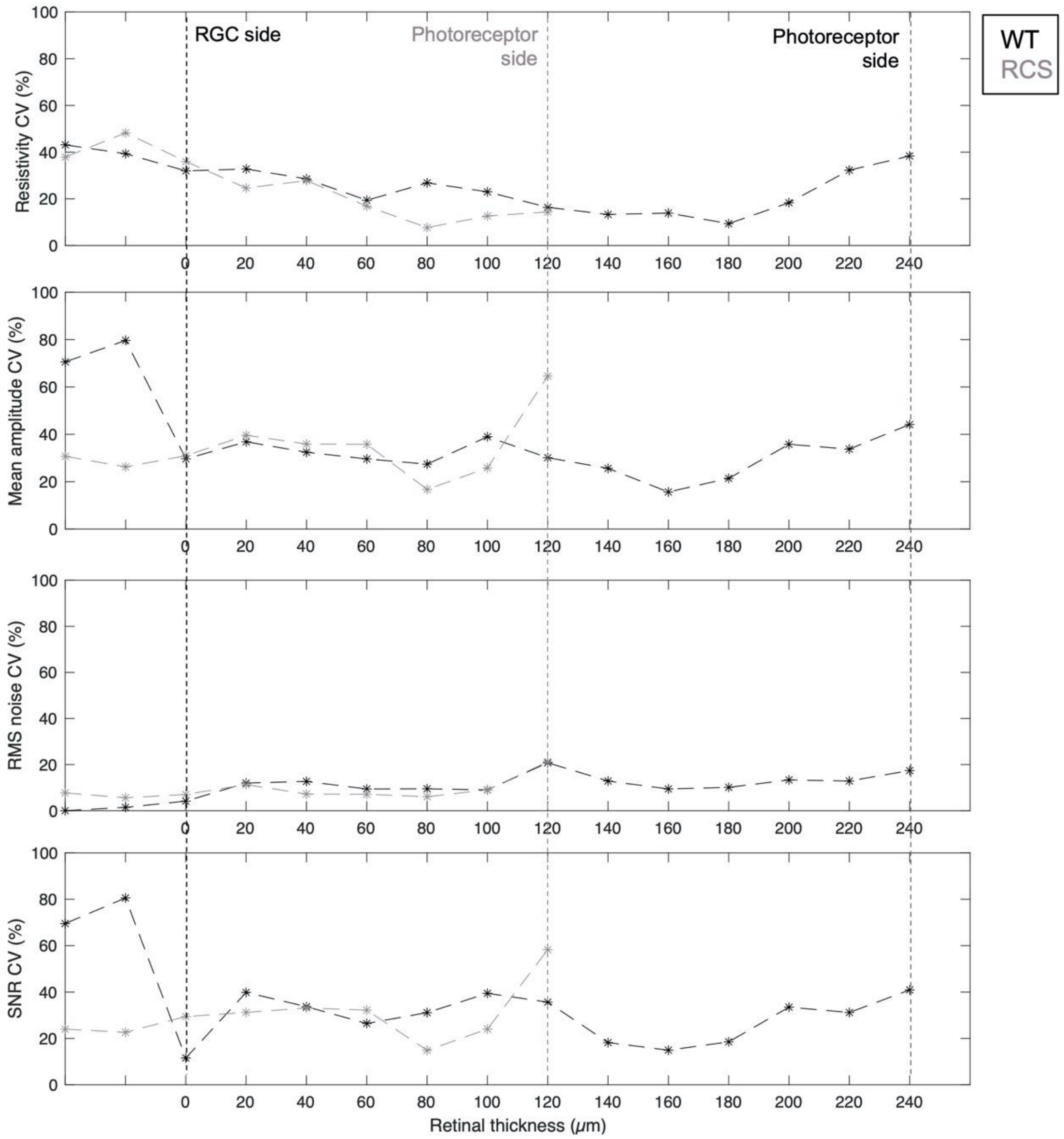

Figure S9: Coefficient of variation (CV) for WT (black) and RCS (grey) rats of the resistivity measurements and features from the electrophysiological data after bootstrapping the data with 1000 samples. Values before 0 μm retinal depth were measured with the electrodes inserted in Ames' medium close to the retinal surface.

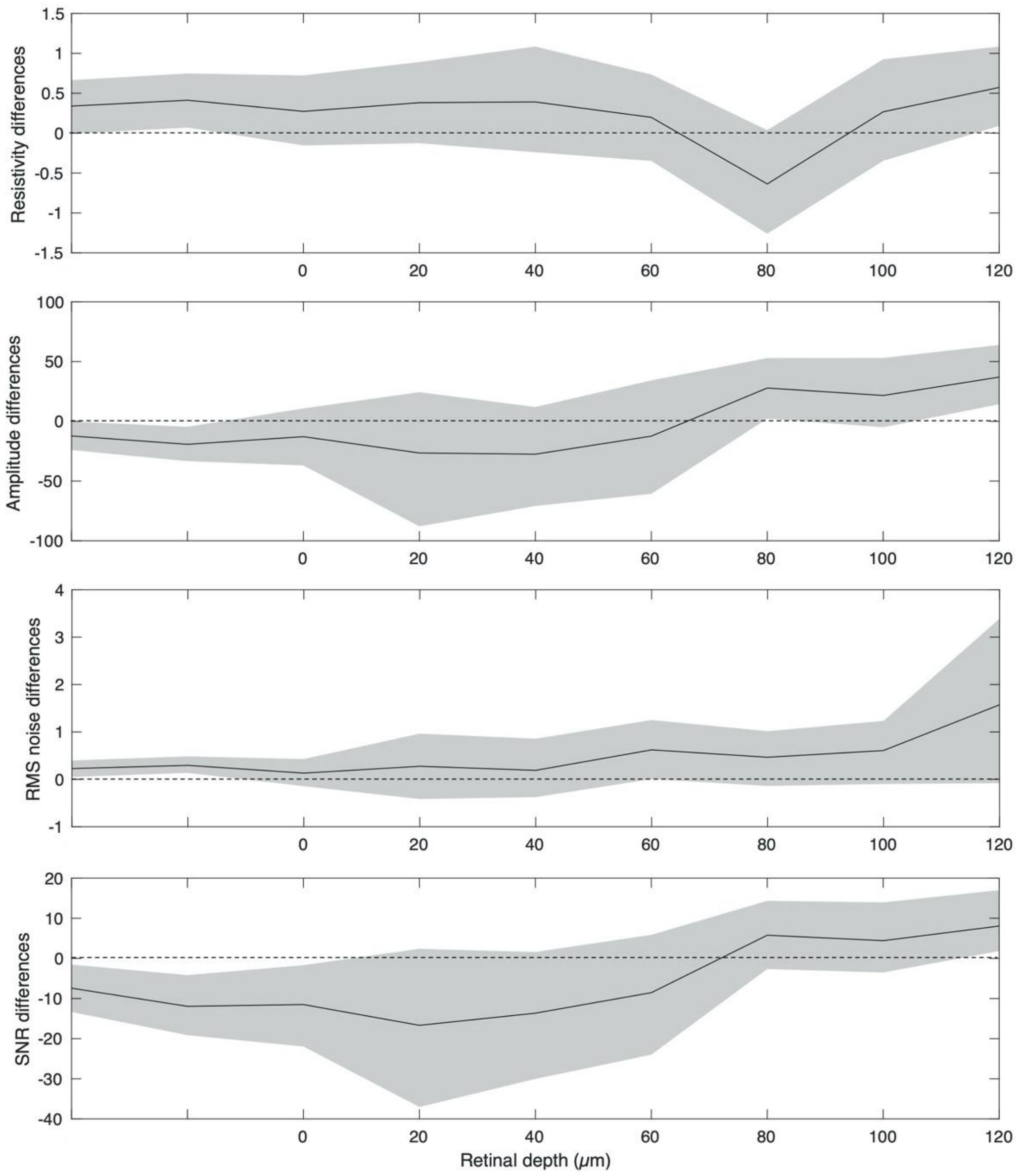

Figure S10: Differences of the means after bootstrapping the WT and RCS data with 1000 samples. The difference of the means is shown with a black line and the 95 % confidence is displayed with a grey patch. Values before 0 μm retinal depth were measured with the electrodes inserted in Ames' medium close to the retinal surface.
